# Comparative staining of *Rhinolophus* spp. white blood cells in blood smears

**DOI:** 10.14202/vetworld.2022.750-756

**Published:** 2022-03-29

**Authors:** Astghik Ghazaryan, Seda Adamyan, Tigran Hayrapetyan, George Papov, Lina Hakobyan, Liana Abroyan, Nane Bayramyan, Sona Hakobyan, Arpine Poghosyan, Hrag Torossian, Zaven Karalyan

**Affiliations:** 1 Department of Zoology, Faculty of Biology, Yerevan State University, A. Manoogian 1, 0025, Yerevan, Armenia; 2 Laboratory of Cell Biology and Virology, Institute of Molecular Biology NAS RA, Yerevan, Armenia; 3 Department of Medical Biology, Yerevan State Medical University after M. Heratsi, Yerevan, Armenia

**Keywords:** blood smears, Rhinolophidae, staining, white blood cells

## Abstract

**Background and Aim:**

A drawback of studies on bat blood smears in the field is the lack of time for fixation because blood sampling using a non-lethal method often provides less time for fixation in smear preparations due to the small volume of blood collected. Usually, there is insufficient blood for another smear preparation, so it is necessary to use blood smears as rationally as possible, especially for rare bats. Many stains are used for staining peripheral blood smears, and they have advantages and disadvantages. This study aimed to examine commonly used stains for blood smears to select the best stain for staining peripheral blood smears in bats.

**Materials and Methods:**

In this study, 48 blood smears of *Rhinolophus* spp. bats were examined using several blood staining methods. Four methods that showed the best results were used in further experiments: Romanowsky-Giemsa, Pappenheim, hematoxylin-eosin, and eosin methylene blue.

**Results:**

Comparative analysis of different methods for staining bat blood smears revealed that the most convenient method for analyzing blood cells is Pappenheim method.

**Conclusion:**

Staining blood smears using Pappenheim method yield the least number of unsuccessful blood smear stains and are quite effective for the morphological analysis of blood cells.

## Introduction

Bats are among the largest and most diverse groups of mammals after rodents, with over 1300 species [1]. They play a significant role in many ecological processes, such as pollination and seed dispersal [2]. Bats are unique in different aspects: They exhibit low reproductive output, exceptional longevity, preference for specific roosting or foraging habitats, colony creation, high sensitivity to climate changes and disturbances, and high positions in trophic webs [3].

In the Caucasus, 35 species of bats that represent 11 genera of three families of the order Chiroptera are registered [4,5]. There are 28-30 species of bats dwelling in Armenia [6,7]. The varied landscapes provide many types of habitats for bats. To date, many cryptic species have been identified in Armenia. Therefore, multisectoral studies (morphological, genetic, ecto-endoparasitic, and physiological) are needed.

Unlike other vertebrate species, the biodiversity of bats is not well studied. There are only a few studies on the blood composition of bats. White blood cell (WBC) investigation is an important tool for studying the immune system status. Recently, studies of bats’ immune systems revealed that bats were an important reservoir for different viruses. Unfortunately, there is a lack of standardization of staining methods for studying the morphology of immune cells and blood smears.

The Romanowsky-Giemsa stain has been used in studies on various blood parasites of bats [8-14]. The Romanowsky-Giemsa stain is also used to show pathological alterations in nucleated blood cells [15]. Other methods are needed to study the morphological features of blood cells such as tetrachrome blood staining [16]. Hematological investigations usually require other types of staining, for example, eosin methylene blue [17], Diff-Quik stain kit modified Romanowsky rapid stain [18], Wright’s stain [19], Pappenheim stain [20,21], and May–Grünwald stain [22,23].

The examination of peripheral blood smears is an important tool for diagnosing the immune system status and hematological disorders. The drawback of studies of blood smears of small wild animals and bats, particularly in the field, is the lack of time for fixation because blood sampling using a non-lethal method often provides less time for fixation in blood smear preparations. This is due to the small volume of blood collected for smears and, as a result, insufficient time for fixation. Usually, there is insufficient blood to prepare another smear; therefore, it is necessary to use blood smears as rationally as possible, especially for rare bats. The blood smear must be well prepared and stained.

Many stains can be used for staining peripheral blood smears, such as the Romanowsky-Giemsa, Pappenheim, hematoxylin-eosin, eosin methylene blue, and tetrachrome. They all have advantages and disadvantages. This study examined several types of commonly used stains for blood smears to select the best stain for staining peripheral blood smears in bats.

## Materials and Methods

### Ethical approval

Bats were captured and sampled according to permission received from the Ministry of Environment of Armenia (2/10.2.7/3457, March 19, 2021). Animal care was done according to the American Veterinary Medical Association Guidelines on Euthanasia and local guidelines for animal care and use (Institutional Review Board/Independent Ethics Committee of the Institute of Molecular Biology of NAS, IRB00004079).

### Study period and location

The study was conducted from March to November 2021. The samples were collected from a cave located in the Vayots Dzor region of Armenia.The samples were processed at Institute of Molecular Biology NAS RA.

### Bat sampling

**Figure-1:**
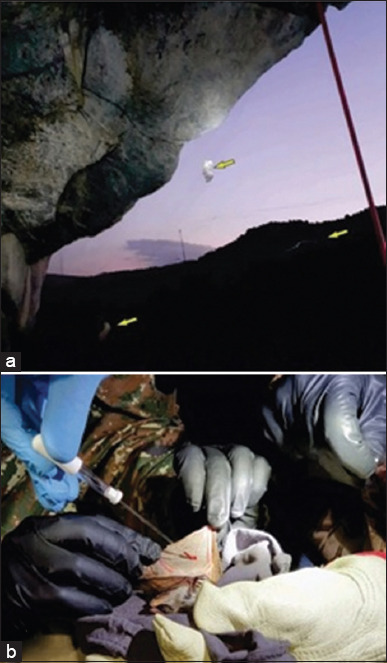
(a) Bats in the net. (b) Taking blood from bats.

Forty-eight blood samples from *Rhinolophus* spp. bats were collected in August 2021. During the day, mist nets were placed in front of the cave. When the bats began to fly in the evening, they were caught in the mist nets. Maternity colonies of horseshoe bats use this cave. By the end of August, the pups could already fly. In our studies, only adult individuals were used. The bats were morphologically identified using taxonomic keys [24]. Bats belonging to the genus *Rhinolophus* (*Rhinolophus euryale*, n=18, and *Rhinolophus ferrumequinum*, n=6; in each case, two blood smears were obtained), were trapped in compliance with the international ethical principles of the Declaration of Helsinki for animal experimentation. Blood sampling was performed using a non-lethal method of tissue sampling [25]. All the bats were released after sampling (Figure-1a).

Bat blood smears were prepared from the blood collected from veins within the uropatagium membrane using a 27-gauge sterile needle (Figure-1b). The peripheral blood smears, stained using different stains, were visually examined using a light microscope (Boeco Germany, BM-800) by two independent and experienced clinical technicians.

### Staining procedure

The standard blood stains Romanowsky-Giemsa (Cypress diagnostics, Belgium), Pappenheim (Cypress diagnostics, Belgium), hematoxylin-eosin (Sigma-Aldrich, Germany), eosin methylene blue (Sigma-Aldrich, Germany), and May–Grünwald (Cypress diagnostics, Belgium) were used for staining the blood smears. Staining was conducted according to the descriptions by Romeis and Kiernan [20,22]. The morphology of the erythrocytes and WBCs was assessed using a Boeco microscope (BM-800, Germany). WBCs (at least 200 in each smear) were counted under oil immersion at ×1200.

## Results

### Main features of bats’ WBCs stained using different staining techniques

Forty-eight blood samples were analyzed from August to October 2021, 36 of which were collected from *R. euryale* and 12 from *R. ferrumequinum*. Several staining methods were used to detect various circulating WBCs using the blood staining techniques. Four showed the best results were Romanowsky-Giemsa, Pappenheim hematoxylin-eosin, and eosin methylene blue. However, the May–Grünwald stain failed to demonstrate acceptable results.

**Figure-2:**
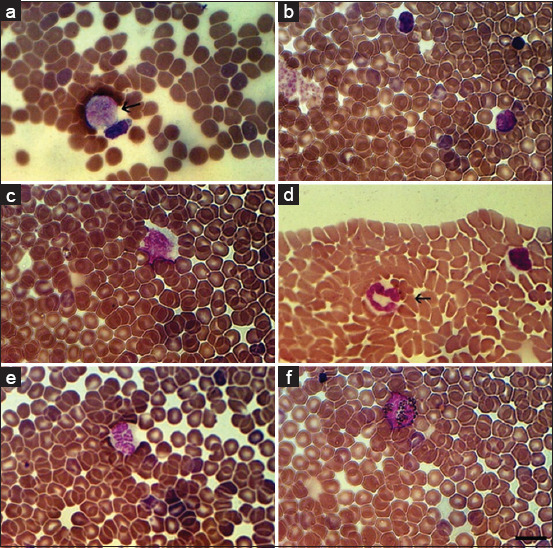
Bats blood smears stained by Romanowsky-Giemsa. (a) Lymphoblast (arrowed); (b) Lymphocytes; (c) Monocyte; (d) Band neutrophil (arrowed); (e) Segmented neutrophil; (f) Eosinophil. Scale bar 10 μm.

Figure-2 presents the data from bats’ blood smears stained using Romanowsky-Giemsa. This staining method easily identifies and is often used for the identification of cells of a blood smear, such as lymphoblasts (Figure-2a), lymphocytes (Figure-2b), monocytes (Figure-2c), band neutrophils (Figure-2d), segmented neutrophils (Figure-2e), and eosinophils (Figure-2f).

**Figure-3:**
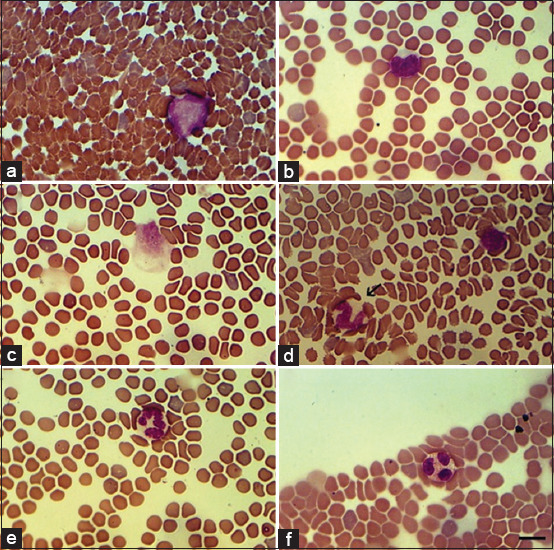
Bats blood smears stained by Pappenheim. (a) Lymphoblast; (b) Lymphocytes; (c) Monocyte; (d) Band neutrophil (arrowed); (e) Segmented neutrophil; (f) Eosinophil. Scale bar 10 μm.

Figure-3 indicates the data of bats’ blood smears stained using Pappenheim’s method. This staining method also easily identifies the cells of a blood smear, such as lymphoblasts (Figure-3a), lymphocytes (Figure-3b), monocytes (Figure-3c), band neutrophils (Figure-3d), segmented neutrophils (Figure-3e), and eosinophils (Figure-3f).

**Figure-4:**
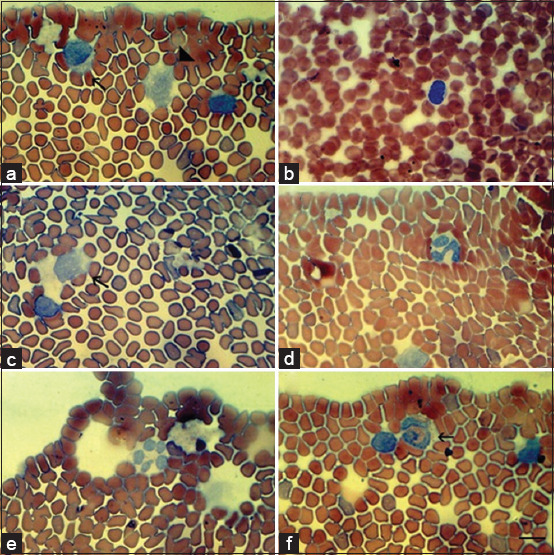
Bats blood smears stained by eosin methylene blue. (a) Lymphoblast (arrowed), monocyte (triangle), and lymphocyte; (b) Lymphocyte; (c) Monocyte (arrowed); (d) Band neutrophil; (e) Segmented neutrophil; (f) Eosinophil (arrowed). Scale bar 10 μm.

In Figure-4, bats’ blood smears stained using eosin methylene blue are illustrated. This stain is sometimes used for the identification of cells of a bats’ blood smear, such as lymphoblasts (Figure-4a), lymphocytes (Figure-4b), monocytes (Figure-4c), band neutrophils (Figure-4d), segmented neutrophils (Figure-4e), and eosinophils (Figure-4f).

**Figure-5:**
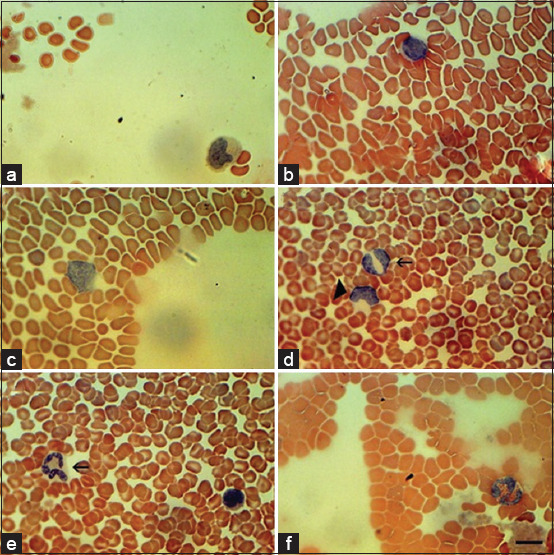
Bats blood smears stained by hematoxylin-eosin. (a) Lymphoblast; (b) Lymphocyte; (c) Monocyte; (d) Band neutrophil (arrowed) metamyelocyte (triangle); (e) Segmented neutrophil; (f) Eosinophil. Scale bar 10 μm.

Figure-5 shows bats’ blood smears stained using hematoxylin-eosin. This stain was used for identifying the main cells of a blood smear, such as lymphoblasts (Figure-5a), lymphocytes (Figure-5b), monocytes (Figure-5c), band neutrophils (Figure-5d), segmented neutrophils (Figure-5e), and eosinophils (Figure-5f).

### Staining

The characteristics and color shades of the main types of leukocytes in bats’ blood smear obtained with all stains are presented in Table-1. Many colors and shades were observed when using the Romanowsky-Giemsa and Pappenheim stains. Eosin methylene blue-stained cells differed mainly in cyan, light blue, and blue (Table-1). Hematoxylin-eosin staining is very different from conventional methods for staining blood smears. It provides an unusual picture of cell structures; many details (azure grains, Auer’s sticks, etc.) are not visible.

**Table-1:** Features and shades of coloring of main types of leukocytes on bats blood smears.

Cell	Cell part	Romanowsky Giemsa	Pappenheim	Eosinmethylene blue	Hematoxylin-eosin
Lymphoblast	Nucleus	Light purple	Light purple	Blue violet	Blue, blue-cyan
	Cytoplasm	Blue, cyan	Light blue	Light blue, cyan	Light orange, pink
Lymphocyte	Nucleus	Purple	Purple	Blue violet	Blue, blue-cyan
	Cytoplasm	Blue	Light blue	Light blue, cyan	Pink, pink gray
Monocyte	Nucleus	Purple, light purple	Purple, light purple	Light blue, cyan	Cyan
	Cytoplasm	Gray-blue	Gray	Gray-blue	Gray
Band neutrophil	Nucleus	Purple or reddish	Purple or reddish-violet	Light blue, cyan	Dark blue
	Cytoplasm	Pink, light pink	Light pink	Light pink	Light reddish, orange
Segmented neutrophil	Nucleus	Purple or reddish	Purple or reddish	Light blue, cyan	Dark blue
	Cytoplasm	Light gray	Light gray	Light pink	Light reddish, orange
Eosinophil	Nucleus	Purple or reddish	Purple or reddish	Light blue, cyan	Dark blue
	Cytoplasm granules	Orange or red-brown	Orange or red-brown	Red, red-brawn	Reddish

Blood samples collected from peripheral vessels of the bat wings were stained using several staining techniques. The best results were obtained using the Romanowsky-Giemsa, Pappenheim, eosin methylene blue, and hematoxylin-eosin stains. Their characteristics are briefly indicated in Table-2. The Romanowsky-Giemsa and Pappenheim stains are the most convenient stains for cell differentiation. However, for fieldwork with small blood volumes obtained using non-lethal sampling, the Pappenheim, hematoxylin-eosin, and eosin methylene blue stains are better options.

**Table-2:** Advantages and disadvantages of different blood staining.

Cell	Romanowsky-Giemsa	Pappenheim	Eosin methylene blue	Hematoxylin-eosin
Fixation	Sensitive	Non sensitive	Non sensitive	Non sensitive
Complexities of coloring	Easily	Moderately	Complex staining procedure double consecutive	Complex staining procedure double consecutive
Blood cell identification	Highly efficient	Highly efficient	Efficient	Efficient
Cytoplasm structures	Easily differentiable	Easily differentiable	Poorly visible	Poorly visible
Nuclear structures	Easily differentiable	Easily differentiable	Easily differentiable	Easily differentiable

### Variations in WBC morphology

**Figure-6:**
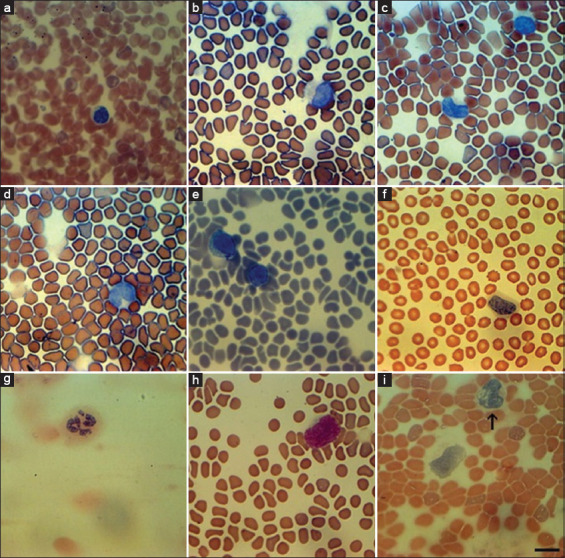
Lymphocyte variation and cells rarely found in blood smears of healthy bats. (a) Small lymphocyte with narrow cytoplasm stained by eosin methylene blue. (b) Large lymphocyte that contain more cytoplasm than small lymphocytes stained by eosin methylene blue. (c) Large lymphocytes oval and elongated nuclei stained by eosin methylene blue. (d) Large lymphocyte with lobular nucleus and narrow cytoplasm stained by eosin methylene blue. (e) Large lymphocytes with narrow and wide cytoplasm stained by eosin methylene blue. (f) Broad cytoplasm lymphocyte stained by hematoxylin-eosin. (g) Hypersegmented neutrophil stained by hematoxylin-eosin. (h) Monoblast stained by Romanowsky-Giemsa. (i) Activated monocyte (arrowed) stained by hematoxylin-eosin.

We studied the morphological characteristics of leukocytes in bats. The leukocyte (mostly lymphocyte) morphology varied markedly within a single population and across the investigated species *R. euryale* and *R. ferrumequinum*. Bat lymphocytes are usually variable in size and shape; they have round to oval or lobular nuclei with condensed chromatin and lighter cytoplasm, with a strong predominance of the nucleus area over the cytoplasm area. Lymphocytes were observed with wide cytoplasm in a less common state (Figure-6). Figure-6g shows hypersegmented neutrophils in bats’ blood smears. In addition, young forms of main WBCs, such as lymphoblasts, and less often monoblasts (Figure-6h) and hyperactive vacuolated monocytes (Figure-6i) were observed.

## Discussion

Usually, 4-5 types of WBCs are present in bats’ blood smears: Lymphocytes, monocytes, neutrophils, eosinophils, and sometimes basophils [22,26,27]. Additional types of WBC were observed in some studies [21,28,29]. Some researchers found it difficult to stain the bat blood smears in field investigations. The deviation of drying blood smears and subsequent artifactual alterations in the morphology of cells leads to the loss of some samples as these staining methods were suboptimal [17]. We also encountered similar problems. The percentage of lost blood smears was the lowest when staining with hematoxylin-eosin, followed by staining with Pappenheim, compared with that when staining with eosin methylene blue. The highest number of lost blood smears was observed when staining using Romanowsky-Giemsa. The major problem of unsuccessful visualization of cells is insufficient staining of the cytoplasm.

The hematoxylin-eosin staining method resulted in the fewest lost smears; however, it was difficult to distinguish between nuclear chromatin and basophilic cytoplasm and between granules and cytoplasm compared with that following Romanowsky staining [30]. A large percentage of bat blood smears were unsuccessfully stained using the Romanowsky-Giemsa method, even though many cell compartments were well distinguishable. The staining problems were not the same for all blood smears. The results of staining were depended on various factors such as the pH of the dye, fixation technique, and variations in the staining time. However, these problems were not permanent and varied greatly. Our results are in consistent with the previous study conducted by Horobin and Walter [30] who stated that the main problems regarding the use of the Romanowsky-Giemsa stain and influences of practical variables such as dye structure, solvent composition, and pH, and of staining time all remain enigmatic.” Another problem with the morphological determination of WBCs is that there is no standard stain for the staining of most types of bat leukocytes.

Comparative analysis of different methods for staining blood smears of bats revealed that the most convenient method for analyzing cell population was staining using the Pappenheim stain. The convenience of this staining method is the low percentage of damaged blood smears because of defective staining compared with all other techniques, except hematoxylin-eosin. Our data also suggest the high efficiency of the determination of morphological characteristics of WBCs in Pappenheim-stained bat smears using image processing.

## Conclusion

Investigations of bats blood smears become necessary in last decade due to their role as virus reservoirs. From the several staining methods used in this study, it can be concluded that staining blood smears using Pappenheim’s method yield the least number of unsuccessful blood smear stains and are quite effective for the morphological analysis of blood cells.

## Authors’ Contributions

AG, SA, TH, and GP: Bats sampling, bats morphological identification, and blood sampling. LH and LA: Carried out blood smears staining. NB, SH, AP, HT, and ZK: WBC analysis. ZK: Analyzed the data and drafted and revised the manuscript. All authors read and approved the final manuscript.
